# Prospective evaluation of inappropriate unable-to-assess CAM-ICU documentations of critically ill adult patients

**DOI:** 10.1186/s40560-015-0119-y

**Published:** 2015-11-26

**Authors:** Kimberly J. Terry, Kevin E. Anger, Paul M. Szumita

**Affiliations:** Department of Pharmacy Services, University of Utah Hospital, 50 North Medical Drive A-050, Salt Lake City, UT 84132 USA; Pharmacy Department, Brigham and Women’s Hospital, 75 Francis Street, Boston, MA 02115 USA

## Abstract

**Background:**

Delirium occurs in the intensive care unit and identification is often performed using a validated assessment tool such as the Confusion Assessment Method for Intensive Care Unit (CAM-ICU) patients. The CAM-ICU has three ratings: positive, negative, and unable to assess (UTA). Patients may often be assigned UTA when it is inappropriate given the level of sedation or medical condition. The purpose of this study is to evaluate the rate of inappropriate UTA CAM-ICU documentations.

**Methods:**

A single-center prospective observational analysis was performed evaluating CAM-ICU documentations from October 27, 2014, to December 26, 2014. Patients admitted to the medical and surgical ICU were included and excluded if admitted to the ICU for less than 24 h. CAM-ICU assessments were performed per institutional guidelines using CAM-ICU scoring as validated in literature. CAM-ICU patient documentations were recorded as positive, negative, UTA, or not assessed. Patients with an appropriate UTA documentation were deeply sedated, non-English speaking, or not medically able to participate in the assessment.

The major endpoint assessed rates of inappropriate UTA CAM-ICU documentations. Minor endpoints evaluated adherence to CAM-ICU documentations and use of pharmacologic agents for symptoms of delirium.

**Results:**

Sixty-one patients were identified with 45 (74 %) medical, 16 (26 %) surgical, of which 27 (44.3 %) were mechanically ventilated. There were 116 UTA documentations with 35 (30.2 %) identified as inappropriate. Of the 906 identified CAM-ICU documentation opportunities, adherence was 439 (48.5 %). Overall, 18 (29.5 %) of the 61 patients were administered pharmacologic agents for delirium management and 5 (27.7 %) had a positive CAM-ICU documented within 24 h.

**Conclusions:**

Rates of inappropriate UTA CAM-ICU documentations may be significantly higher than reported in literature. Additional research is needed to identify an acceptable rate of inappropriate UTA CAM-ICU assessments and its clinical impact on delirium management.

## Background

Delirium is an independent risk factor for increased morbidity and mortality in critically ill patients and is often unrecognized [1–3]. The 2013 Society of Critical Care Medicine pain, agitation, and delirium practice guidelines recommend routine monitoring of delirium using a validated assessment tool [2]. Critical care nurses and ICU providers are often in frontline positions to identify, assess, and document symptoms of delirium [4, 5]. As ICUs develop protocols to implement routine delirium screening, limited information exists regarding appropriateness of individual ratings and acceptable margins of error [6].

Mixed results from observational studies question whether routine bedside assessments are reliable and accurate in identifying delirium in both intubated and non-intubated critically ill patients [7–9]. The Confusion Assessment Method for the Intensive Care Unit (CAM-ICU) is a validated screening tool to identify delirium and has three ratings: positive, negative, and unable to assess (UTA) [10]. Characteristic features of delirium include impairments in short-term memory, disorientation, inattention, and fluctuating course [11]. The criteria for an UTA rating are a Richmond Agitation Scale Score (RASS) of −4 to −5, neurologic impairment or underlying dementia, and inability to adequately perform the assessment due to language or hearing barriers [12]. The objective of this study was to evaluate the rate of inappropriate UTA CAM-ICU documentations within our institution’s medical and surgical ICU populations.

## Methods

Our study was approved by the Brigham and Women’s Hospital Investigational Review Board. Individual patient consent was not required as no intervention outside standard of care was being performed. This single-center prospective cohort analysis evaluated the rates of inappropriate UTA CAM-ICU documentations. An appropriate UTA assessment was defined as patients with a corresponding RASS of −4 or −5 and physical limitations to performing a CAM-ICU, such as being unable to squeeze the assessor’s hand, documented history of neurological dysfunction, hearing impaired, or non-English speaking. Any patient who was assessed as UTA without any of the defined parameters was considered to have an inappropriate UTA. Inappropriate positive and negative documentations were also collected. For patients who received positive or negative CAM-ICU assessments, but had a documented physical inability to squeeze the assessor’s hand, history of neurological dysfunction, hearing impairment, or non-English speaking was considered to have an inappropriate positive or negative assessment.

Our institution has routinely used CAM-ICU assessments since 2006 in patients able to participate. Nurses assess and identify four features of ICU delirium which include level of sedation, acute onset, inattention, and disorganized thinking. Our institution provides advanced level ICU nursing care with 1:1 to 1:2 nurse to patient ratio, monthly education, and skills teaching. Nurses are evaluated routinely for ICU competency and are an integral part of ICU protocol development and implementation of delirium assessment. The assessment tool is not limited to mechanically ventilated patients and is utilized in all ICU patients. Each ICU has an electronic and paper version of the institutional CAM-ICU protocol. The first step in the protocol asks the assessor the patient’s current level of sedation. If a patient has a RASS of −4 or −5, the protocol directs the assessor to document UTA. If the patient has a RASS of −3 to +4, the assessor will continue to determine the patient’s mental status, inattention, and disorganized thinking through as series of questions. Part of the protocol asks the patient to squeeze the assessor’s hand for every letter “A” in the word SAVEAHAART. If patients are unable to perform this task, they are assessed with a UTA assessment. In addition, our institution does not have the CAM-ICU readily available for patients who do not speak English, and therefore, a CAM-ICU cannot be assessed and patients should be assessed with a UTA designation.

The data collection period from October 27, 2014, to December 26, 2014, included critically ill adult patients from the surgical and medical intensive care units (SICU, MICU) with at least one documented CAM-ICU assessment. A convenience sample of SICU patients from October 27, 2014, to November 21, 2014, and MICU patients from November 23, 2014, to December 26, 2014, Monday through Friday were enrolled. A single pharmacist assessed CAM-ICU ratings, and thus a convenience sample was performed to fit the covering pharmacist’s perspective units during the collection period. Patients were excluded from enrollment if admitted to the ICU for less than 24 h.

Our institution guidelines recommend a CAM-ICU be assessed and documented at least every 8 h for each patient. CAM-ICU and RASS documentations were collected daily during the collection period along with use of atypical antipsychotics or sleep aids indicated for delirium. A critical care pharmacist collected data each day throughout the study period recording documented data as well as observing patient assessments as a part of daily rounding responsibilities. CAM-ICU documentations were categorized as one of four options: UTA, positive, negative, or not assessed. After collecting the documented CAM-ICU assessment, appropriateness was determined by our defined patient criteria. Not assessed was assigned to an undocumented CAM-ICU assessment.

### Primary endpoint

The primary endpoint evaluated the rates of inappropriate UTA CAM-ICU documentations as defined by the study criteria.

### Secondary endpoint

Secondary endpoints reported overall adherence to CAM-ICU documentations, inappropriate positive or negative CAM-ICU documentations, and use of pharmacologic agents for symptoms of delirium. Adherence was defined as the number of CAM-ICU assessments documented over the total CAM-ICU documentation opportunities.

### Statistics

From a study by Swan, the observed rates of inappropriate UTA documentations before and after an education campaign were 32 and 19 %, respectively [13]. Based on these results, we estimated inappropriate UTA documentation rates greater than or equal to 25 % to be considered significant. A chi-square test, Student’s *t* test or Mann-Whitney *U* test was used where appropriate.

## Results

During our study period, 67 patients were identified. Six patients were excluded for an ICU stay less than 24 h, leaving 61 patients included in the final analysis with 116 unique UTA CAM-ICU documentations (Table 1).

**Table 1:** Baseline demographics

Baseline demographics	*N* = 61
Age- mean±SD	64 ± 15.8
Male n, (%)	34 (55.7)
APACHE II- median [IQR]	19 [13–27]
Medical, *n* (%)	45 (74 )
Respiratory failure	20 (44.4)
Sepsis	11 (24.4)
DKA	4 (8.9)
Other	10 (22.2)
Surgical, *n* (%)	16 (26)
GI	4 (25)
Thoracic	3 (18.7)
Spinal	2 (12.5)
ENT	1 (6.25)
Urologic	1 (6.25)
Other	5 (31.25)
CAM-ICU exclusions, n	
Dementia	2
Physical limitation	1
Non-English speaking	2
MV, n (%)	27 (44.3)
Median ICU LOS (days), [IQR]	3.4 [1.5–9.8]
Median hosp LOS (*n* = 60) (days), [IQR]	11 [5.2–20.4]
Death, n (%)	11 (18)

*DKA* diabetic ketoacidosis, *GI* gastrointestinal, *ENT* ear, nose, throat, *MV* mechanical ventilation, *LOS* length of stay

### Primary endpoint

A total of 35 (30.2 %) of the 116 UTA assessments documented were inappropriate (Table 2). Based on our study definitions, this was a significantly high rate of inappropriate UTA observations. The SICU had a greater number of inappropriate UTA documentations than the MICU, 55 and 25 %, respectively (*p* = 0.014). All UTA CAM-ICU assessments were deemed inappropriate due to a documented RASS between −3 and +4 (Table 3).

**Table 2:** Rates of inappropriate CAM-ICU assessments

CAM-ICU	MICU	SICU	Total	*p* value
UTA	96	20	116	
Inappropriate UTA, *n* (%)	24 (25)	11 (55)	35 (30.2)	0.0142
Positive	90	13	103	0.0629
Inappropriate positive, *n* (%)	20 (22.2)	7 (53.8)	27 (26.2)	0.0369
Negative	168	52	220	0.0293
Inappropriate negative, *n* (%)	4 (2.4)	8 (15.4)	12 (5.5)	0.0014
Total CAM-ICU assessments	354	85	439	
Not assessed	283	184	467	<0.0001

**Table 3:** UTA CAM-ICU and corresponding sedation scores

RASS	UTA CAM-ICU assessments (*n* = 35)
−5	0
−4	0
−3	6
−2	6
−1	3
0	18
1	2
2	0
3	0
4	0

### Secondary endpoint

Total adherence to CAM-ICU documentation was 439 (48.5 %) of the 906 documentation opportunities. Of the 467 missed documentations, the majority occurred on the overnight shifts, 170 (36.4 %), then evening shifts 135 (28.9 %). Inappropriate positive or negative documentations were observed in 39 assessments (Table 4). Overall, 18 (29.5 %) of the 61 patients used pharmacologic agents for symptoms of delirium. Five of the eighteen patients (27.7 %) had an appropriate CAM-ICU positive rating within 24 h prior to administration of a pharmacologic agent for documented symptoms of delirium.

**Table 4:** Inappropriate positive and negative CAM-ICU documentations

Reason for inappropriate assessment	No. of assessments
Non-English speaking	14
Dementia	10
Physical limitations	15

## Discussion

Our analysis highlights a significant rate of inappropriate UTA documentations using the CAM-ICU, suggesting that real-world use of the assessment tools in a medical and surgical ICU environment has potential for inappropriate documentation. The 2013 Society of Critical Care Medicine management guidelines recommend routine assessment and documentation of delirium using one of two validated tools, the Intensive Care Delirium Screening Checklist (ICDSC) or CAM-ICU [2]. Focusing on the CAM-ICU, minimal literature is available describing rates of inappropriate UTA documentation.

Inappropriate UTA documentations were due to a corresponding RASS score between −3 and +4. A study by Swan described pre- and post-educational interventions to improve UTA CAM-ICU documentations. Factors contributing towards the number of inappropriate UTA assessments were from misinterpretation of a patient’s depth of sedation [13]. Performing a quality control project in the future to address any educational gaps in sedation assessment may help reduce the number of inappropriate UTA documentation.

We observed 39 inappropriate CAM-ICU positive or negative documentations. Swan observed rates of inappropriate positive and negative CAM-ICU assessments, and after an educational intervention, no statistical differences were reported [13]. Similarly, we observed inappropriate documentations between all CAM-ICU ratings available. Documentation errors may be due to multiple reasons. A study by Voyer et al. assessed the accuracy of nursing documentation using the CAM, a 10-symptom domain. Within the 10 domains, 69.1% to 100 % of symptoms were not documented, due primarily to education gaps of interpreting symptoms of delirium [7]. We found the MICU had statistically less inappropriate UTA, positive, and negative documented assessments compared to the SICU. The varying patient populations between the MICU and SICU as well as differences in the frequency of CAM-ICU education may contribute to culture differences surrounding ICU delirium assessment and interpretation. Education for nurses as well as the medical team occurs frequently at the bedside during patient rounds covering various topics based upon the patient population. In the MICU, delirium management is often integrated into daily bedside discussions, whereas the SICU utilizes monthly didactics to review delirium assessment primarily for the medical team. These culture differences were not assessed in our study; however, these differences cannot be ruled out as a contributing factor in CAM-ICU documentation differences. Within our analysis, education gaps, errors in documentation, or misinterpretation may explain the large number of inappropriately documented CAM-ICU assessments in addition to the number of patients administered pharmacologic agents for delirium who did not have a positive CAM-ICU assessment. Other studies have suggested that the use of consistent terminology and interdisciplinary involvement may reduce the incidence of delirium misinterpretation [14, 15].

To improve overall adherence in routine CAM-ICU assessments, educational programs similar to Swan and Pun et al. may need to be implemented [13, 16]. Both used on site teaching and learning programs with nurses modeling appropriate CAM-ICU assessments. Although Swan was able to significantly decrease rates of inappropriate UTA CAM-ICU documentations, Pun et al. improved overall delirium detection and documentation [16]. Our institution has been using the CAM-ICU as a delirium assessment tool for many years. From our results, ICU teams may benefit from a multidisciplinary education initiative to decrease the number of inappropriate UTA CAM-ICU documentations through proper patient identification.

### Limitations

Limitations of this study are its design being observational with no tested intervention. As a prospective observational study within a single center, it may be difficult to apply our observations to other ICU environments. Validation of each CAM-ICU assessment was not critiqued by an expert rater due to limited resources and therefore was left to strict definitions. Rates of missed assessments were greatest on the overnight and afternoon shifts. This may be related to minimizing nighttime awakenings during the overnight shifts to promote sleep and patients off the unit for procedures in the afternoon. Our study did not assess reasons for missed documentations and reported only if they were not documented. Lastly, our study was not designed to assess if inappropriate UTA documentations lead to worse outcomes such as increased morbidity or mortality, therefore leaving to question the clinical impact of an inappropriate UTA CAM-ICU documentation.

## Conclusions

Rates of inappropriate UTA CAM-ICU documentations may be significantly higher than reported in literature. Additional research is needed to identify an acceptable rate of inappropriate UTA CAM-ICU assessments and its clinical impact on delirium management.

### Key messages

- CAM-ICU unable-to-assess ratings may occur frequently in real-world evaluations.
- Acceptable rates of inappropriate CAM-ICU assessments have not been well defined in literature.
- Inappropriate CAM-ICU assessments have unknown clinical impact and may warrant further research.
